# DNA vaccine constructs against enterovirus 71 elicit immune response in mice

**DOI:** 10.1186/1479-0556-5-6

**Published:** 2007-04-19

**Authors:** Wong Siew Tung, Sazaly Abu Bakar, Zamberi Sekawi, Rozita Rosli

**Affiliations:** 1 Dept. of Human Growth and Development, Faculty of Medicine and Health Sciences, Universiti Putra Malaysia, 43400 Serdang, Selangor, Malaysia; 2 Department of Medical Microbiology, Faculty of Medicine, Universiti Malaya Medical Center, 50603 Kuala Lumpur, Malaysia; 3 Dept of Clinical Laboratory Sciences, Faculty of Medicine and Health Sciences, Universiti Putra Malaysia, 43400 Serdang, Selangor, Malaysia

## Abstract

**Background:**

Enterovirus 71 (EV71) is a major causative viral agent responsible for large outbreaks of hand, foot and mouth disease (HFMD), a common rash illness in children and infants. There is no effective antiviral treatment for severe EV71 infections and no vaccine is available. The objectives of this study were to design and construct a DNA vaccine against Enterovirus 71 using the viral capsid protein (VP1) gene of EV71 and to verify the functionality of the DNA vaccine *in vitro *and *in vivo*.

**Methods:**

The VP1 gene of EV71 from two local outbreak isolates were amplified using PCR and then inserted into a eukaryotic expression vector, pVAX1. The 3.9 kb recombinant constructs were transformed into competent *E. coli *cells and the positive clones were screened and selected using PCR analysis, restriction digestion analysis and DNA sequencing. The constructs were then tested for protein expression in Vero cells. Subsequently, in the *in vivo *studies, female Balb/c mice were immunized with the DNA vaccine constructs. Enzyme Linked Immunosorbent Assay (ELISA) and virus neutralizing assay were performed to detect the presence of anti-VP1 IgG in mice and its neutralizing effect against the EV71.

**Results:**

The pVAX1 vector was successfully cloned with the VP1 gene from each of the isolate (S2/86/1 and 410/4) in the correct orientation and in-frame. The DNA vaccine constructs with the VP1 gene were shown to be expressed in a cell-free *in vitro *expression system. The VP1 protein was successfully expressed in the mammalian cell line and was detected using RT-PCR, Indirect Immunofluorescence Assay (IFA) and western blotting. The anti-VP1 IgG levels in mice immunized with the DNA vaccine constructs increased after the first booster but declined following the second booster. The anti-VP1 IgG in the mice immunized with the DNA vaccine constructs exhibited neutralising activity against EV71.

**Conclusion:**

The promising results obtained in the present study have prompted further testing to improve the expression and immunogenicity of this potential EV71 DNA vaccine.

## 1. Background

Enterovirus 71 (EV71) belongs to the genus of enteroviruses from the family *Picornaviridae*. It possesses a single stranded RNA genome of approximately 7500 nucleotides of positive polarity, which is encapsulated in a capsid containing 60 copies of each of the four structural proteins, VP1 through VP4 [1]. The antigenic diversity among the enteroviruses is caused by variations within capsid proteins VP1 to VP3, but neutralization epitopes are most densely clustered on VP1 [2].

Enterovirus 71, along with coxsackievirus A16 (CA16), is a major causative viral agent responsible for large outbreaks of hand, foot and mouth disease (HFMD), a common rash illness in children and infants. EV71 is thought to spread by contact with fecal contaminated materials. Infection by the virus is often asymptomatic or may manifest as mild self-limiting illness which is often characterized by the presence of characteristic lesions on the palms, soles and oral mucosa. EV71 and CA16 are genetically closely related. However, unlike CA16 that is more limited in its pathogenicity to HFMD, EV71 is also associated with severe complications involving the central nervous system (CNS) such as aseptic meningitis, encephalitis and poliomyelitis-like paralysis [3,4].

Since the first report of EV71 infection which occurred in California in 1969 [5], world-wide reports of outbreaks have followed. The neurovirulence of EV71 first came to attention in 1975 in Bulgaria when 44 people died of a polio-like disease [6]. Epidemics of EV71 causing CNS diseases subsequently occurred in New York, Australia, Europe and Asia [7-10]. An unusual epidemic of HFMD complicated by fatal myocarditis and pulmonary edema occurred in Malaysia in 1997, and EV71 had been implicated as the etiology of the outbreak [11]. Thirty-one children in Sarawak, Malaysia and four children in Peninsular Malaysia succumbed to the infection within hours of admission to the hospitals [12]. The largest EV71 epidemic reported to date occurred from April to December of 1998 in Taiwan in which a variety of clinical manifestations were observed. These included HFMD, encephalitis, meningitis, herpangina and poliomyelitits-like paralysis. In this outbreak, more than 90,000 children infected with HFMD were reported. Among these patients, more than 320 children were hospitalized with suspected meningitis, encephalitis, or acute flaccid paralysis, and at least 55 died, suggesting neurovirulence of the pathogen [13].

There is no effective antiviral treatment for severe EV71 infections and no vaccine is available. Thus, the only current means to prevent EV71 infection is through avoidance of contact between infected and susceptible individuals [14]. Since no effective antiviral agents are available, the need for an effective EV71 vaccine is urgent to immunize the public before an outbreak occurs. A formalin-inactivated EV71 vaccine was developed in response to the Bulgarian epidemic in 1975 [6,15] but was not used to control the epidemic and has not been used since. Thus, no data on the efficacy of the Bulgarian vaccine is available. Recently, several candidates of EV71 vaccines using different approaches are being investigated. These include formalin-inactivated whole virus vaccine, DNA vaccine and recombinant protein vaccine. These vaccine constructs remain promising vaccine strategies that require further refinement, thus further study and development are required [16-18].

In this study, a DNA vaccine encoding the VP1 gene of EV71 was designed and constructed. This vaccine candidate was tested *in vitro *for expression of VP1 protein in mammalian cell culture and followed by *in vivo *testing of the ability for the protein to be expressed and to elicit an immune response in mice.

## 2. Materials and methods

### 2.1 Construction and validation of DNA vaccine

The VP1 gene of EV71 isolate 410/4 (genotype B4) and EV71 isolate S2/86/1 (genotype B4) which have been cloned into pCR^® ^Blunt Vector (Zero Blunt™ PCR Cloning Kit, Invitrogen) were provided by Prof. Mary Jane Cardosa, Universiti Malaysia Sarawak (UNIMAS). The VP1 gene of both isolates was amplified by polymerase chain reaction (PCR). A forward primer 5'-GATCTGCAGGCCACC**ATG**GGGGATAGAGTGGCAGATG-3' and a reverse primer 5'-GAAGCGGCCGC**CTA**AAGGGTAGTAATGGCAGTACG-3' were used to amplify the VP1 gene of EV71 isolate 410/4, whereas a forward primer 5'-GATCTGCAGGCCACC**ATG**GGAGATAGAGCGGCAGATG-3' and a reverse primer 5'-GAAGCGGCCGC**CTA**AAGGGTAGTAATGGCAGTACG-3' were used to amplify the VP1 gene of EV71 isolate S2/86/1. The *Pst *I site and *Not *I site (underlined) were included in the forward and reverse primers, respectively for the purpose of cloning. The PCR temperature profile included denaturation at 95°C for 5 min, followed by 30 cycles of denaturation at 95°C for1 min, annealing at 62°C and extension at 72°C for 1 min 20s, followed by 10 min of heating at 72°C. The PCR products were then purified and subjected to automated sequencing for confirmation. Subsequently, the amplified VP1 gene was digested with *Pst *I and *Not *I and ligated with the pVAX1 vector. The pVAX1/VP1 plasmid was transformed into One Shot^® ^TOP 10 Chemically Competent Cells (Invitrogen).

### 2.2 Detection of cell-free in vitro expression of VP1 protein

The Transcend™ Non-Radioactive Translation Detection Systems (Promega, U.S.A.) was used to detect the VP1 proteins synthesized *in vitro*. Biotinylated lysine residues are incorporated into nascent proteins during translation in a cell-free single-tube, coupled transcription/translation reaction for eukaryotic *in vitro *protein expression. After running SDS-PAGE and electroblotting, the biotinylated VP1 proteins can be visualised by binding to Streptavidin-Horseradish Peroxidase, followed by chemiluminescent detection.

### 2.3 In-vitro protein expression in eukaryotic cells

Vero cells (ATCC CCL-81) were cultured in RPMI 1640 with 10% fetal calf serum added until growth reach 80% confluence. Lipofectamine™ 2000 (Invitrogen) was used to transfect the DNA vaccine constructs following the manufacturer's instruction. The negative control experiment was performed using the backbone vector, pVAX1. The transfected cells were incubated at 37°C in a 5% CO_2 _incubator for 48 hours before detection of VP1 gene expression was carried out.

### 2.4 Detection of VP1 mRNA

The expression of VP1 gene was determined by detecting the presence of VP1 mRNA using RT-PCR. Isolation of total RNA from the transfected cultured cells was performed using SV Total RNA Isolation System (Promega). RETROscript™ kit (Ambion) was used to reverse transcribe the extracted RNA into its complementary DNA sequence (cDNA). The cDNA was used as template for the amplification of VP1 gene using PCR method as described in 2.1. The PCR products were subjected to agarose gel electrophoresis to detect the presence of VP1 gene.

### 2.5 Indirect Immunofluorescence Assay (IFA)

Light Diagnostic Enterovirus 71 Monoclonal Antibody (Chemicon International, CA, USA) was used to detect the VP1 protein expressed in cell culture using an indirect immunofluorescence assay (IFA). Briefly, the transfected cells were removed after 48 hours of incubation and resuspended in PBS to make cell spots on microscope slides. The slides were fixed in chilled (2–8°C) acetone for 10 minutes. Sufficient Enterovirus 71 Monoclonal Antibody was added to cover the cells on the slides and incubated at 37°C for 30 minutes in an incubator. The slides were washed gently with PBS and then added with sufficient anti-mouse IgG: FITC (Chemicon International, CA, USA) to cover the cells. Subsequently, the slides were mounted with cover slips and examined using a fluorescence microscope (DMRA2, Leica).

### 2.6 Western blot

After 72 hours of incubation, the transfected cells were harvested and protein isolation was carried out using Mammalian Protein Extraction Reagent (Pierce, U.S.A.). The protein samples were loaded onto a discontinuous SDS-polyarylamide gel with 5% stacking gel and 12.5% separating gel. Electrophoresis was performed using Mini-PROTEAN^® ^III Electrophoresis Cell (Bio-Rad, U.S.A.). Subsequently, the protein bands were electrophoretically transferred from the SDS-polyacrylamide gel to PVDF membrane using a Mini Trans-Blot^® ^Electrophoretic Transfer Cell (Bio-Rad, U.S.A.). The VP1 protein was detected using rabbit anti-EV71 polyclonal antibody and alkaline phosphatase conjugate chicken anti rabbit secondary antibody (Chemicon, U.S.A.).

### 2.7 DNA vaccination of BALB/c mice

Ethical approval to work with animals was obtained from the Animal Care and Use Committee (ACUC), Faculty of Medicine and Health Sciences, UPM. Female Balb/c mice aged between 6–8 weeks with 19–21 gm weight were divided in groups of 6 and were immunised by intramuscular injection with each of the DNA vaccine constructs: pVAX1/VP1-4, pVAX1/VP1-S or pEGFPN-1/VP1 (EV71 VP1-expresing vector which was provided by Prof. Dr. Sazaly Abu Bakar, University of Malaya and served as a control). The mice were injected with 100 μl of the plasmid DNA solution in phosphate buffered saline (PBS) at a concentration of 100 μg per mouse. The two negative control groups, each with 6 mice, were injected with the backbone vector pVAX1 (100 μg per mouse) and PBS (100 μl per mouse). The animals were given food and water *ad lib*. The mice in each group were given booster injections at 14 and 28 days post-immunisation. Blood samples were collected from the mice by cardiac puncture at day 0, day 14, day 28 and day 42 of experiment.

### 2.8 Enzyme Linked Immunosorbent Assay (ELISA)

To detect the presence of anti-VP1 IgG in the sera of immunised mice, Enzyme Linked Immunosorbent Assay (ELISA) was performed using ELISA Reagent Kit (Chemicon International, U.S.A.) and 96-well microtiter plates coated with VP1 fusion protein and S. tag protein (negative control). Optical density (O.D.) was read at 450 nm using an ELISA reader. The adjusted O.D. of each sample was calculated by subtracting the mean O.D. of the control well from the mean O.D. of VP1 antigen well. Statistical analysis of the adjusted O.D. between the different groups immunized with DNA vaccines and negative controls was performed using one-way ANOVA test.

### 2.9 Virus neutralizing assay

The virus neutralisation test was performed to assess the ability of the mice serum to neutralise the live enterovirus 71. A positive serum from an EV71-infected child (provided by Prof. Dr. Sazaly Abu Bakar, University Malaya) was used as a positive serum control in this test. The test was carried out using the pooled mice sera of each group (pVAX1/VP1-S, pVAX1/VP1-4, pVAX1 and PBS) collected on day 14. Vero cells were seeded in 96-well plates at a density of 1 × 105 cells/well in RPMI supplemented with 10% FBS and incubated overnight. To perform the neutralisation test, the mice sera samples were first incubated at 56°C for 30 minutes to inactivate the complement. A two-fold serial dilution (from 1:2 to 1:64) was carried out by diluting the test mice serum with RPMI medium without FBS. Subsequently, 80 μl of each diluted test serum sample was added with 80 μl of 1 × 10^3 ^p.f.u. of live EV71 (1:1 ratio) and incubated for 1 hour at 37°C. All the tests were performed in triplicates. Then, the Vero cells were washed with 1 × PBS and incubated with 50 μl inoculum of test serum-virus mixture per well at room temperature for 1 hour. Virus control wells were included as negative controls in the test where the cells were incubated with only live virus that have not been treated with any serum. After incubation, the cells were added with 200 μl of RPMI supplemented with 5% FBS per well and incubated in cell culture CO_2 _incubator at 37°C with 5% CO_2 _for 3 days. The cytopathic effect (CPE) of cells and the number of plaques formed were monitored under an inverted tissue culture microscope (Olympus CK40, Japan). The titre was read as the highest dilution that resulted in more than 50% CPE.

## 3 Results

### 3.1 Construction of DNA vaccine

**Figure 1:**
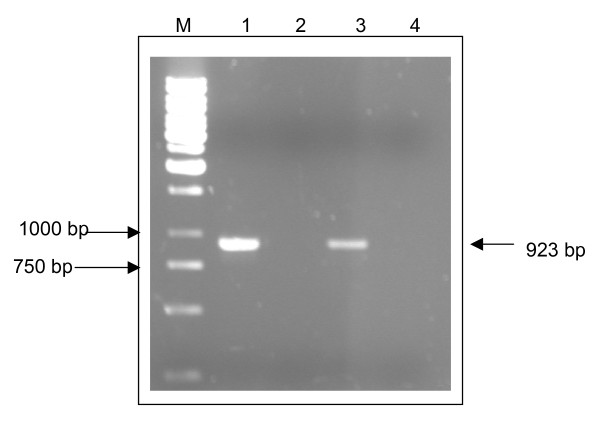
**Amplification of VP1 genes of EV71 isolate S2/86/1 and isolate 410/4 by PCR**. Lane M shows a 1 kb molecular marker (Fermentas). Lane 1 and lane 3 each shows a band of size 923 bp, which represent the PCR product of VP1 gene of EV71 isolate S2/86/1 and isolate 410/4, respectively. Lane 2 and lane 4 which show no band of PCR product are PCR negative controls that used water as template.

The amplification of VP1 gene for both isolates produced a 923 bp band as shown in Figure 1. Each amplicon (VP1 gene) had been designed to include the *Pst *I site and *Not *I site at both ends to facilitate cloning into expression vector, pVAX1 (Invitrogen, U.S.A.). The PCR products were purified using QIAquick Gel Extraction Kit (Qiagen, Germany) and sent for sequence analysis. The sequencing results confirmed that the amplicons are the VP1 gene of EV71.

The VP1 gene was successfully cloned into the eukaryotic expression vector, pVAX1 and transformed into One Shot^® ^TOP 10 Chemically Competent Cells (Invitrogen). The positive clones that carried the pVAX1/VP1 DNA vaccine were screened and selected using PCR and restriction digestion analysis. The desired constructs were further confirmed by automated DNA sequencing. The pVAX1 vector that was successfully cloned with VP1 gene of each EV71 isolate S2/86/1 and isolate 410/4, in the correct orientation and in frame were designated as pVAX1/VP1-S and pVAX1/VP1-4, respectively.

**Figure 2:**
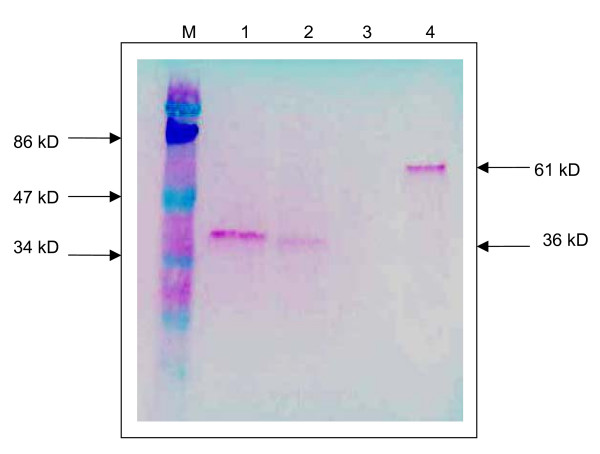
**Western blot analysis on eukaryotic cell-free *in vitro *expression of VP1 Protein using Transcend™ Chemiluminescent Translation Detection Systems (Promega, U.S.A.)**. Lane M shows a prestained protein molecular weight marker (Fermentas, Lithuania). Lane 1 to lane 4 represent the eukaryotic cell-free *in vitro *transcription/translation reactions of pVAX1/VP1-S, pVAX1VP1-4, pVAX1 and Luciferase T7 control DNA respectively. Lane 1 and lane 2 each shows a band of size 36 kD, indicating the translated VP1 protein. Lane 3 shows no band of translation product with using backbone vector pVAX1 alone. Lane 4 shows a band of size 61 kD, indicating the translated luciferase protein, which serve as a positive control for the eukaryotic cell-free *in vitro *expression reactions.

The DNA vaccine constructs were verified for VP1 gene expression using a cell-free *in vitro *expression system, TNT^® ^Quick Coupled Transcription/Translation System (Promega. U.S.A.). The biotinylated lysine residues were incorporated into the VP1 protein during translation. Using the chemiluminescent detection method with streptavidin-alkaline phosphatase in Western blotting, a protein band with a size of 36 kD was obtained in each transcription/translation reaction of pVAX1/VP1-S and pVAX1/VP1-4 which indicated the translated VP1 protein (Figure 2, lanes 1 and 2). There was no protein band observed in the reaction sample of backbone vector pVAX1 (Figure 2, lane 3) since it did not contain any coding sequence.

### 3.2 Detection of VP1 mRNA using RT-PCR

**Figure 3:**
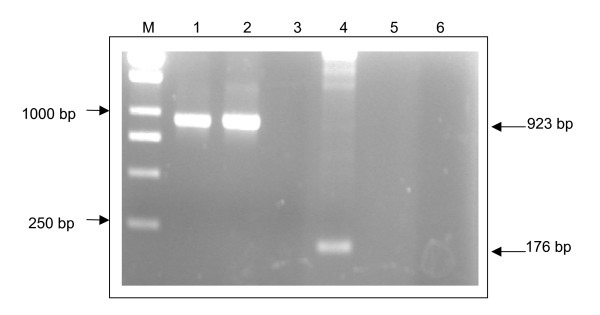
**Amplification of VP1 gene by RT-PCR**. Lane M shows a 1 kb molecular weight marker (Fermentas, Lithuania). Lane 1 and lane 2 each shows a band of size 923 bp, indicating the amplified VP1 gene from extracted mRNA of Vero cells transfected with pVAX1/VP1-S and pVAX1/VP1-4 respectively. Lane 3 shows no RT-PCR product amplified from extracted mRNA of Vero cells transfected with backbone vector pVAX1 alone, which serve as a negative control. Lane 4 shows the PCR product of size 176 bp when backbone vector pVAX1 was amplified using T7 forward primer and BGH reverse primer. Lane 5 and lane 6 show no PCR product generated from the extracted mRNA of Vero cells transfected with pVAX1/VP1-S and pVAX1/VP1-4 respectively, when using T7 forward primer and BGH reverse primer.

A PCR product of expected size 923 bp that represents the VP1 gene was obtained using cDNA derived from Vero cells transfected with pVAX1/VP1-S and pVAX1/VP1-4 (Figure 3, lanes 1 and 2, respectively). To eliminate the possibility that the VP1 PCR product was produced because of plasmid DNA contamination, T7 forward primer and BGH reverse primer were used to amplify the cDNA derived from cells transfected with pVAX1/VP1. No product was amplified (Figure 3, lanes 5 and 6) since the T7 forward and BGH reverse priming sites were only found on pVAX1 vector but not the cDNA that was derived from the transfected Vero cells. Plasmid DNA pVAX1 was used as a control for this PCR reaction using T7 and BGH primers. A PCR product of 176 bp was obtained (Figure 3, lane 4), indicating that this primer pair could produce an amplicon from the plasmid DNA pVAX1. No PCR product was obtained using cDNA derived from cells transfected with control plasmid, the backbone vector pVAX1 (Figure 3, lane 3).

### 3.3 Indirect Immunofluorescence Assay (IFA)

**Figure 4:**
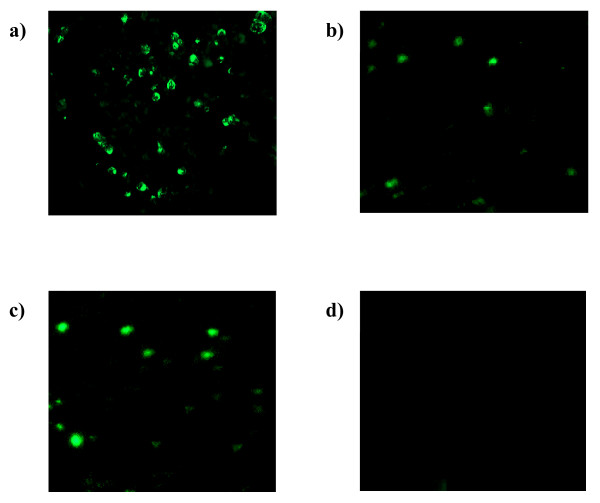
**Detection of VP1 protein using indirect immunofluorescence assay (IFA)**. Expression of VP1 protein in transfected vero cells at 48 hours post-transfection was detected by using indirect immunofluorescence assay (IFA). Figure a) shows the EV 71 positive control slide. Figure b) and c) show vero cells transfected with pVAX1/VP1-S and pVAX1/VP1-4, respectively. Figure d) shows vero cells transfected with pVAX1.

Enterovirus 71 positive and negative control slides (Chemicon, U.S.A.) were included to assure proper performance of the test. The positive control slide showed cells exhibiting apple-green fluorescence (Figure 4a). Apple-green fluorescence signals were detected in the cells transfected with pVAX1/VP1-S and pVAX1/VP1-4 (Figure 4b and 4c, respectively). The cells transfected with the backbone vector pVAX1, which served as a negative control did not exhibit any fluorescence (Figure 4d).

### 3.4 Western blot

**Figure 5:**
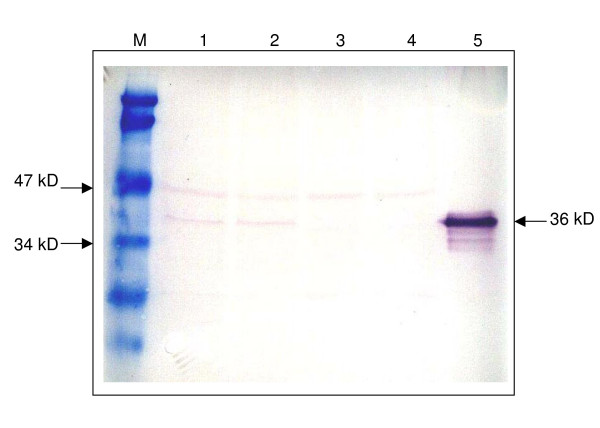
**Western blot analysis of transfected vero cells at 72 hours post transfection using rabbit anti-VP1 polyclonal antibody**. Lane M shows a prestained protein molecular weight marker (Fermentas, Lithuania). Lane 1 and lane 2 each shows a band of size 36 kD, which represent the expressed VP1 protein in Vero cells transfected with pVAX1/VP1-S and pVAX1/VP1-4 respectively. Lane 3 and lane 4 represent Vero cells transfected with backbone vector pVAX1 alone and non-transfected Vero cells respectively, show no band of size 36 kD, indicating the absence of VP1 protein. Lane 5 represents the extracted protein of EV 71, shows a band of size 36 kD, which serve as a positive control.

A band of 36 kD was visualised in protein extracts from cells transfected with pVAX1/VP1-S and pVAX1/VP1-4, which represents the VP1 protein (Figure 5, lanes 1 and 2, respectively). No visible band parallel to the VP1 protein (36 kD) was observed in the cells transfected with backbone vector pVAX1 (Figure 5, lane 3) and non-transfected vero cells (Figure 5, lane 4), which indicated the absence of VP1 protein in these two samples that served as negative controls. Extracted protein of enterovirus 71 that served as a positive control produced a 36 kD band of VP1 protein (Figure 5, lane 5).

### 3.5 Enzyme Linked Immunosorbent Assay (ELISA)

**Figure 6:**
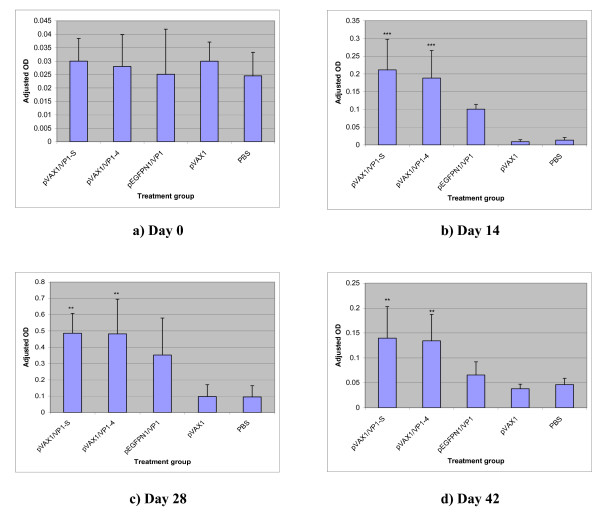
Adjusted OD at 450 nm of ELISA for anti-VP1 IgG of mice serum collected at different days post-immunization at 1:50 dilution.

The anti-VP1 IgG elicited in immunised mice sera on day 0, day 14, day 28 and day 42 post-vaccination was measured using ELISA. There was no significant difference (p > 0.05) between the anti-VP1 IgG responses elicited in all the mice groups before vaccination, i.e. on day 0 (Figure 6a). The anti-VP1 IgG elicited by pVAX1/VP1-S and pVAX1/VP1-4 increased to a very significant level (p < 0.001) on day 14 when compared to the negative control groups (Figure 6b). After the first booster, the anti-VP1 IgG in these two groups continually increased to a level with the highest OD value (Figure 6c) but subsequently declined after the second booster although the level was still statistically significant (p < 0.01) compared to negative control groups (Figure 6d).

There was no significant increase (p > 0.05) in anti-VP1 IgG level of mice immunized with pEGFPN1/VP1 on day 14 when compared with the level in negative control groups (Figure 6b). The anti-VP1 IgG level of this group rose after the first booster, but it was not statistically significant (p > 0.05) when compared to the negative control groups (Figure 6c). The level dropped following the second booster (Figure 6d).

**Figure 7:**
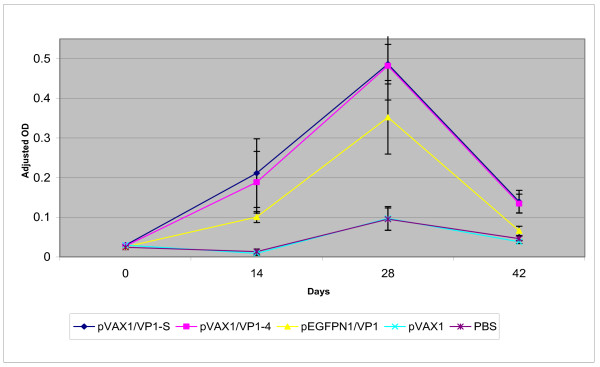
Adjusted OD at 450 nm of ELISA for anti-VP1 IgG of mice serum collected at different time points at 1:50 dilution.

Overall, the anti-VP1 IgG level in all the three DNA vaccine groups (pVAX1/VP1-S, pVAX1/VP1-4 and pEGFPN1/VP1) increased after the first booster but declined following the second booster (Figure 7). A statistically significant difference between the level of anti-VP1 IgG elicited in DNA vaccine groups (pVAX1/VP1-S and pVAX1/VP1-4) and the negative control groups (pVAX1 and PBS) at day 14 (p < 0.001), day 28 (p < 0.01) and day 42 (p < 0.01) was observed. However, the pEGFPN1/VP1 group did not show any statistically significant difference (p > 0.05) compared to the negative control groups.

### 3.6 Virus neutralisation assay

The virus neutralisation test was used to evaluate the ability of the DNA vaccine constructs, pVAX1/VP1-S and pVAX1/VP1-4 to elicit a neutralising antibody response. By adding live virus to the test serum, the presence of neutralising antibody in the test serum will bind to the virus. If this occurs, no virus residue will remain to infect the Vero cells. On the other hand, if there is no neutralising antibody in the test serum, no antibody will bind to the virus and the virus will then infect the cells, resulting in cytopathic effect (CPE). It has been reported that cells infected with EV71 exhibited a CPE with morphological changes, including rounding up, shrinkage, cytoplasmic blebbing, nuclear condensation and detachment from the culture dish, indicative of apoptotic cells [19]. Similar CPE was observed in this study.

**Figure 8:**
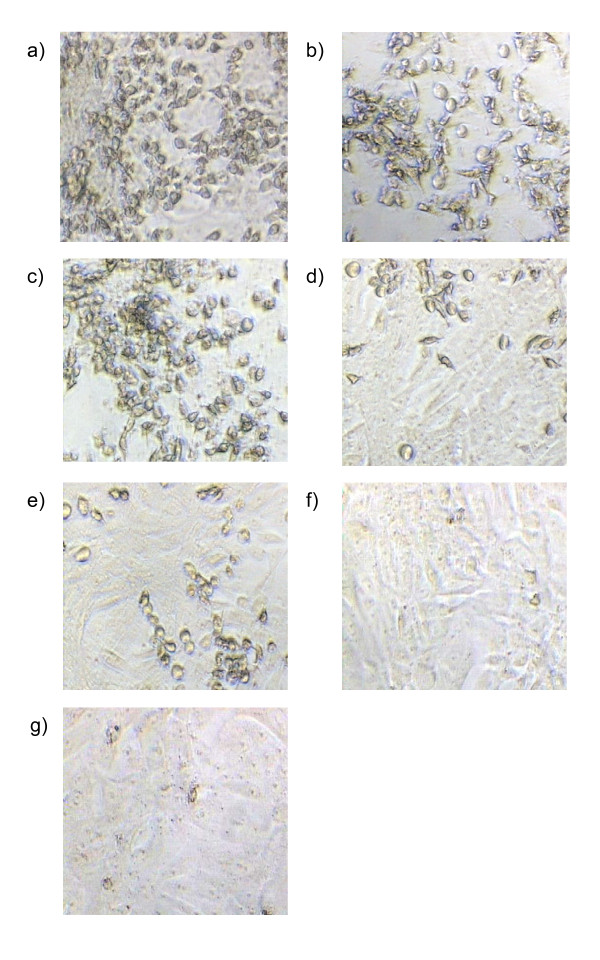
**Virus neutralisation test of immunized mice seraon day 14**. The neutralising activity of immunised mice sera against EV71 on day 14 was evaluated by virus neutralisation test using Vero cells. Figure 8a) and 8b) show almost 100% of CPE in Vero cells resulted by sera of mice immunized with pVAX1 and PBS (negative) respectively. Figure 8c) represents the virus control well with almost 100% CPE observed. Figure 8d) and 8e) showed the neutralizing activity of sera of mice immunized with pVAX1/VP1-S and pVAX1/VP1-4 respectively, where approximately 50% CPE was observed. Figure 8f) showed almost 100% neutralization of EV71-infected human positive serum where no CPE was observed. Figure 8g) represents the normal Vero cells that are not infected with EV71. All the dilution of serum samples in Figure 8a) to 8f) was at 1:32 and microscopy examinations were carried out at 100× magnification. Arrows indicate the CPE.

After 3 days of incubation, all the virus control wells showed 100% CPE (Figure 8c). Meanwhile, the positive EV71 infected human serum resulted in almost 100% neutralisation at the dilution of 1:8 to 1:64, where no sign of CPE was observed, hence demonstrating the neutralising effect (Figure 8f). At the dilution of 1:2 and 1:4, the human serum had caused cell death without signs of CPE where it might be due to the side effect of high serum concentration.

**Figure 9:**
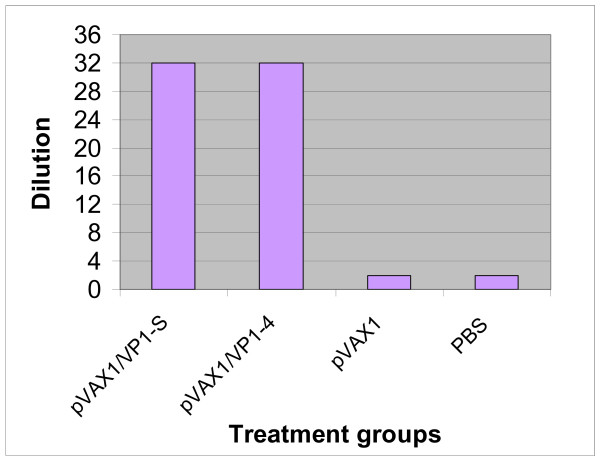
**The highest dilution of mice sera that resulted inmore than 50% CPE**. The sera of DNA vaccine groups (pVAX1/VP1-S and pVAX1/VP1-4) had resulted in approximately 50% CPE at a dilution of 1:32. Almost 100% CPE was observed in the sera of negative control groups (pVAX1 and PBS) at the dilution of 1:2 and the subsequent dilutions.

The sera of DNA vaccine groups (pVAX1/VP1-S and pVAX1/VP1-4) had resulted in approximately 50% CPE at a dilution of 1:32 (Figure 8d and 8e). Only about 15% and 10% CPE was observed at the dilution of 1:16 and 1:8, respectively. Obviously, the positive EV71 infected human serum antibody reduced EV71 infectivity much more effectively, compared with the sera of animals immunized with DNA vaccines. The sera of negative control groups, pVAX1 (Figure 8a) and PBS (Figure 8b), showed no neutralization activity where almost 100% CPE was observed at all dilutions. The highest dilution of mice sera that resulted in more than 50% CPE is shown in Figure 9.

## 4 Discussion

In the present study, the DNA vaccine encoding the VP1 gene of Enterovirus 71 (EV71) was designed and constructed. These DNA vaccine constructs were verified and tested for *in vitro *expression of VP1 gene in Vero cell line. VP1 mRNA was detected in transfected cell culture by RT-PCR whereas the presence of VP1 protein was confirmed by indirect immunofluorescence assay (IFA) and western-blotting. VP1 mRNA in transfected Vero cells was detected by reverse transcription-polymerase chain reaction (RT-PCR) at 48 hours post-transfection. However, the presence of VP1 mRNA is just a qualitative data and it cannot reveal the level of VP1 expression, as the copy number of target transcript was not determined. Real-time PCR analysis can be conducted in order to quantify the *in vitro *expression level of VP1 gene in cell culture if necessary. The VP1 protein expressed in cell culture was successfully detected using Indirect Immunofluorescence Assay (IFA) and Western blot method at 48 hours and 72 hours post-transfection, respectively. The presence of VP1 protein could not be detected with Western blotting at time points earlier than 72 hours post-transfection, where this could be due to low level of expression and thus insufficient quantity of protein that can be detected. Comparatively, Western blotting using colorimetric method is not as sensitive as IFA, which utilises the aid of an optical instrument (fluorescence microscope) for detection of target protein. There are many possible reasons for low expression of an antigen, such as toxicity, poor codon usage, or mRNA structure instability. It is also possible that the antigenic protein is unstable and rapidly degraded [20]. The successful *in vitro *expression implies that the constructs were able to direct VP1 protein synthesis correctly in eukaryotic cells, thus confirming the functionality of the constructs.

In order to assess the immune responses to DNA vaccine, immunisation of female Balb/c mice was carried out using intramuscular injection of the naked plasmid. Following DNA immunisation, the antigenic proteins expressed from plasmids especially for viral antigens seem to be expressed, folded and assembled in the same manner as when they are expressed during a natural viral infection. As a result, the antigens are genuine with all the conformational epitopes required for protection being expressed [21,22].

One of the major barriers to the development of an EV71 vaccine is the lack of a suitable animal model for the testing of vaccine immunogenicity and efficacy. Laboratory mice are only susceptible to EV71 infection in the first 4 days of life and become completely resistant by 6 days of age [18,23]. EV71 infection caused no apparent clinical symptoms in all strains of adult mice tested, including BALB/c, C3H, ICR, CD28 knock-out, and TNF-α receptor knock-out mice [16]. Therefore, if further studies were to be carried out in order to assess the protection against lethal EV71 infection provided by the antibody elicited following DNA vaccination, newborn mice have to be challenged with enterovirus 71 and then followed by passive transfer of maternal antibody from immunised dams to the newborn mice.

In the present study, the results of ELISA have confirmed the immunogenicity of DNA vaccine constructs in eliciting immune response *in vivo *with the presence of specific anti-VP1 IgG in mice sera. Studies have shown that expression of genes from plasmids following injection into muscles is optimal at 7–14 days post-injection [24]. In this study, anti-VP1 IgG elicited by pVAX1/VP1-S and pVAX1/VP1-4 increased to a very significant level on day 14 after the first dose of injection. Following the first booster, the anti-VP1 IgG in these two groups continued to increase to a level with the highest OD value (Figure 6) but subsequently declined after the second booster. The decline of IgG levels observed in this study might have been accompanied by an increase in cytolitic T cells. Tanghe *et al*. [25] demonstrated that the antibody levels in mice immunised with tuberculosis DNA vaccine decreased after the second booster injection while an increase in IFN-γ and IL-2 levels was detected. This observation showed a shift towards the cellular immune response, which may provide an explanation to the decline of anti-VP1 IgG levels observed in the present study. However, this has to be confirmed by further investigation.

From the results of the virus neutralisation assay, the anti-VP1 IgG in the mice immunised with DNA vaccine constructs exhibited neutralising activity against EV71. However, the neutralising effect of EV71-infected human positive serum was demonstrated to be much higher compared with the sera of animals immunised with DNA vaccines. The possible explanation for this might be the immune system of mice immunised with DNA vaccine was only exposed to the antigenic epitopes that are found in VP1 protein of EV71 while the EV71-infected human had been exposed to all the various antigenic epitopes of the whole EV71 virus particle. Therefore, the neutralising antibodies produced by human immune system are able to identify the wide range of various antigenic epitopes of EV71 and thus possess higher neutralising effect. Another possible reason could be that the confirmation of the protein synthesized by the plasmids may differ from that in the live virus particles. Therefore, minor differences in the confirmation of the antigenic epitopes could result in different neutralisation titres [26]. In this study, only mice sera of day 14 post-immunisation (after a single dose of vaccination) were tested for the neutralising activity, so there is no further data to show the neutralisation titre after the first and second booster vaccination. However, studies have shown that neutralising antibodies were developed after a first single-dose of DNA vaccination with the neutralisation titre elevated and persisted for an extended interval following booster injections [27-29]. Wu *et al*. (16) had reported that the neutralisation titre of DNA vaccinated mice was still detectable at 32 weeks post-immunisation and it also maintained a titre similar to the one that immunised with inactivated virus.

pEGFPN1/VP1 which was provided by Prof. Dr. Sazaly Abu Bakar (University Malaya) served as a positive control in the vaccination experiment of the present study. This construct has been shown to express VP1 protein in mice after vaccination. The anti-VP1 IgG level of this group did not show any statistically significant difference compared to the negative control groups. It might be due to the fact that the pEGFPN1 is not an expression vector that is specifically designed for DNA vaccination use, such as the pVAX1 vector. Hence, pEGFPN1/VP1 is not as efficient as pVAX1/VP1-S and pVAX1/VP1-4 in eliciting immune response in mice.

Development of transgenic mice expressing the appropriate human receptor molecule(s) for enterovirus 71 would be the best and promising approach in order to understand the pathogenesis of enterovirus encephalitis and to test the candidate vaccines. This approach has been extremely successful for poliovirus [30]. Since the receptor for EV71 remains unknown, identification of the EV71 receptor is a major priority in vaccine research, as it will allow the development of a transgenic mouse model for studies of EV71 pathogenesis and vaccine efficacy.

## 5 Conclusion

Overall, this study has given promising results on genetic immunisation with DNA vaccine encoding VP1 gene of EV71. With further study and improvement, use of the DNA vaccine might be a potential vaccine strategy against EV71.
